# A phase II prospective study of the “Sandwich” protocol, L-asparaginase, cisplatin, dexamethasone and etoposide chemotherapy combined with concurrent radiation and cisplatin, in newly diagnosed, I/II stage, nasal type, extranodal natural killer/T-cell lymphoma

**DOI:** 10.18632/oncotarget.16334

**Published:** 2017-03-17

**Authors:** Ming Jiang, Li Zhang, Li Xie, Hong Zhang, Yu Jiang, Wei-Ping Liu, Wen-Yan Zhang, Rong Tian, Yao-Tiao Deng, Sha Zhao, Li-Qun Zou

**Affiliations:** ^1^ Department of Medical Oncology, State Key Laboratory, Cancer Center, West China Hospital of Sichuan University, Chengdu, China; ^2^ Radiation Oncology of Cancer Center, West China Hospital of Sichuan University, Chengdu, China; ^3^ Pathology Department, West China Hospital of Sichuan University, Chengdu, China; ^4^ Department of Oncology, Dujiangyan Medical Center, Dujiangyan, Sichuan, China; ^5^ Department of Nuclear Medicine, West China Hospital of Sichuan University, Chengdu, China

**Keywords:** nasal-type, extranodal NK/T cell lymphoma, L-asparaginase, cisplatin, etoposide and dexamethasone (LVDP)

## Abstract

Nasal-type, extranodal NK/T cell lymphoma (ENKTCL) is a special type of lymphomas with geographic and racial specificity. Up to now, the standard first-line treatment is still not unified. In our previous report, the “sandwich” protocol produced good results. Continuing to use the “sandwich” mode, a new chemotherapy composed of L-asparaginase, cisplatin, etoposide and dexamethasone (LVDP) plus concurrent chemoradiotherapy (CCRT) was conducted in more patients with newly diagnosed, I/II stage ENKTCL. The results showed that 66 patients were enrolled. Overall response rate was 86.4% including 83.3% complete response and 3.0% partial remission. With the median follow-up of 23.5 months, 3-year overall survival and 3-year progression-free survival were 70.1% and 67.4%, respectively. The survival rate in stage II and extra-cavity stage I was significantly less than that in limited stage I (***p*** < 0.05). Therefore, we thought that the “sandwich” mode was worthy of being generalized and LVDP combined with CCRT was an effective protocol for I/II stage ENKTCL. But this regimen was not suitable for all stage I/II patients and warrants larger sample and layering investigation. This study was a registered clinical trial with number ChiCTR-TNC-12002353.

## INTRODUCTION

Nasal-type, extranodal NK/T cell lymphoma (ENKTCL) is a special type of non-Hodgkin's lymphoma, which is rare in Europe and North America, but relatively common in Asia and South America [1, 2]. In China, the incidence of ENKTCL is higher and secondary to diffuse large B cell lymphoma, accounting for about 11% of all lymphomas [3]. About 60-90% of ENKTCL derive from nasal cavity and its adjacent sites and up to 75% of patients have I/II Ann Arbor stage at the initial diagnosis [4, 5]. However, traditional therapy based on anthracyclines achieved unsatisfied effect—5-year overall survival (OS) rate is less than 50% [2, 6]. Chemotherapy composed of non-anthracycline drugs showed superior effect [7–9], especially L-asparaginase-based regimens which are the mainstream choice. But the optimal chemotherapy, the way of chemotherapy combined with radiotherapy and the dose of radiation are still undefined. In our previous report, the “sandwich” protocol, 2 cycles of systemic chemotherapy made up of L-asparaginase, vincristine and prednisone (LVP) were followed by sequential radiotherapy with 56Gy and supplemented with 2-4 cycles of LVP, achieved good improvement and mild side effects [10] indicated that the “sandwich” protocol deserves further research in I/II stage ENKTCL patients.

In order to improve the efficacy and further study on “sandwich” regimen, we designed and administrated a new chemotherapy regimen including L-asparaginase (L-ASP), cisplatin, etoposide and dexamethasone (LVDP) combined with concurrent radiation and cisplatin. A prospective clinical study was conducted in more patients with newly diagnosed, I/II stage ENKTCL. The primary end points of this study: (1) short term efficacy (overall response rate (ORR), complete response (CR), (2) 3-year progress-free survival (PFS) and OS. The secondary endpoints: short term side effects.

## RESULTS

### Basic characteristics

Sixty-six ENKTCL patients with newly diagnosed, stage I/II were enrolled. Basic characteristics were shown in Table 1. The median age was 41.5y (rang: 13 to 70y). There were 44 male patients with 2.0 times as many as the female (22 cases). 14 cases (21.2%) were in limited IE stage, 29 (43.9%) in extra-cavity IE stage and 23 (34.8%) in IIE stage. Twenty- five cases (37.9%) had B symptom. Twenty- three (34.8%) had regional lymph nodes invasion. According to the NK/T cell lymphoma international prognostic index (NKIPI) [11], 25 patients (37.9%) were 0 score, 22 patients (33.3%) were 1 score, 16 patients (24.2%) were 2 score and 3 patients (4.5%) were 3 score.

**Table 1:** Basic characteristics of the patients

Characteristics	Number of patients (%)
**Age, y (median, range, 41.5 (13-70))** <60 ≥60	58 (87.9) 8 (12.1)
**Sex** Male Female	44 (66.7) 22 (33.3)
**ECOG Score** 0 1 2	45 (68.2) 20 (30.3) 1 (1.5)
**Stage** Limited IE Extra-cavity IE IIE	14 (21.2) 29 (43.9) 23 (34.8)
**Serum LDH** Normal Increase	47 (71.2) 19 (28.8)
**“B” symptom** No Yes	41 (62.1) 25 (37.9)
**IPI** 0 1	41 (62.1) 25 (37.9)
**NKIPI** 0 1 2 3	25 (37.9) 22 (33.3) 16 (24.2) 3 (4.5)
**Regional lymph nodes metastasis** No Yes	43 (65.2) 23 (34.8)
**Perforation** No Yes	60 (90.9) 6(9.1)
**Platelet count** Normal Decrease Increase	51 (77.3) 4 (6.1) 11 (16.7)
**Lymphocyte count** Normal Decrease Increase	52 (78.8) 12 (18.2) 2 (3.0)
CSWOG staging* I II III	18 (27.3) 26 (39.4) 22 (33.3)

ECOG, Eastern Cooperative Oncology Group; LDH, Lactic dehydrogenase; IPI, International Prognostic Index; NKIPI, NK/T-cell lymphoma International Prognostic Index;

*Extranodal NK/T-cell lymphoma, nasal-type new staging system by Chinese Southwest Clinical Oncology Group (CSWOG) (http://meetinglibrary.asco.org/content/128647-144)

### Response

**Figure 1:**
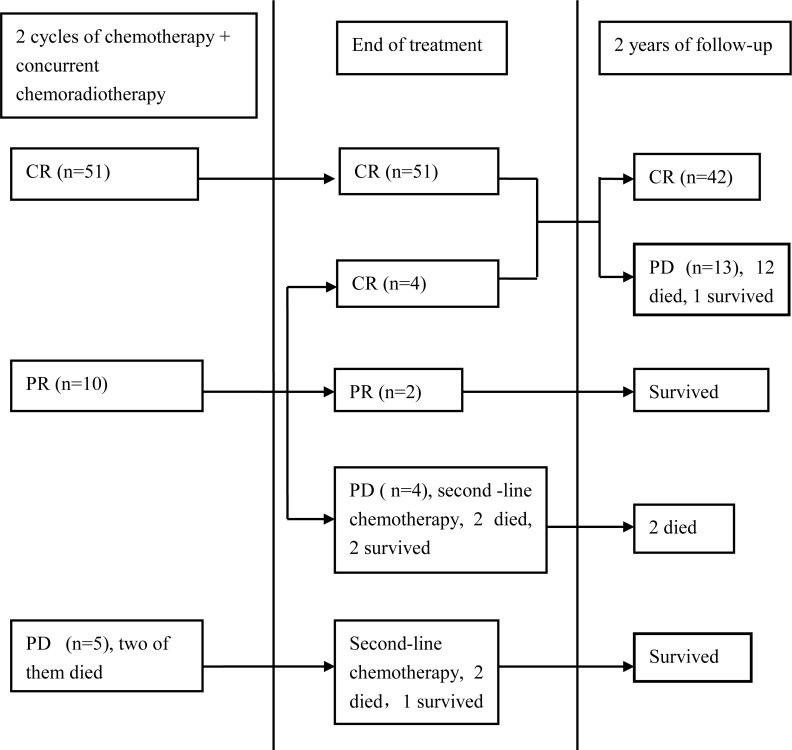
Treatment response and prognosis in patients (CR complete response, PR partial response, PD progressive disease).

A total of 336 cycles of chemotherapy (4.8 cycles in average, 1-6 cycles) were administrated, pegaspargase was used in 50 cycles of them. 59 patients (89.4%) received 4-6 cycles of chemotherapy. 7 patients (10.6%) had 1-3cycles of chemotherapy. Chemotherapy was stopped in one patient due to the onset of pancreatitis after 3 cycles. Two refused to continue chemotherapy after 3 courses. 4 patients' treatments, because of disease progression, were terminated, 2 of whom died soon and 2 received second-line therapy. The patients completed radiotherapy according to schedule, except the two died early. After two-cycle chemotherapy and CCRT, 51 cases had CR (77.3%), 10 had partial response (PR) (15.2%) and the ORR was 92.4%. At the end of therapy, 55 had CR (83.3%), 3 had PR (3.0%) and the ORR was 86.4%. These were shown in Figure 1.

### Survival

**Figure 2:**
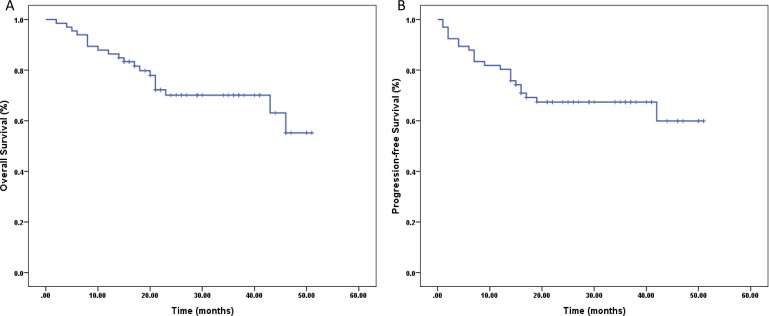
Survival curves A., Overall survival for all patients. B., Progression-free survival for all patients.

The 66 patients were followed up until April 2015. As shown in Figure 1, 13.6% (9/66) experienced disease progression during the therapy period including 1 local progression and 8 system failure. 13 of 55 (23.6%) patients with CR experienced recurrence. Among of them, two patients (3.6%) experienced relapses in the previous radiation field, and eleven (20%) experienced systemic recurrence, with five in skin soft tissue (one with pulmonary metastasis and one with liver and bone metastasis), two in bone marrow (one with splenic metastasis), one in lung, two in gastrointestinal tract and one in the central nervous system. Only one of these thirteen patients survived at the deadline of follow-up, living with stable condition after second-line chemotherapy. Eleven patients didn't achieve CR after first-line treatment. Up to the end of follow-up, 8 of them died (8/11, 72.7%) and 3 (3/11, 27.3%) survived. With the median follow-up of 23.5 months (range: 12 to 51 months), a total of 22 (33.3%) had disease progression including 4.5% for local failure and 28.8 % for systemic failure. For patients who had disease progression, their median survival was 15 months. For whole group, the 3-year OS rate was 70.1% (Figure 2A), and the 3-year PFS rate was 67.4% (Figure 2B), the median OS had not been reached.

### Toxicity

As shown in Table 2, the most common toxicity was that patients were allergic to L-ASP (45.5%). Among of them, 3.0% with negative skin test underwent slight allergic reaction including skin rash and itching, and recovered soon after stopping the infusion, administrating hormone and calcium. Hematological toxicities were also common during chemotherapy, but most of them were mild. 11 patients (16.7%) had grade 3 or 4 leukopenia and 2 patients (3.0%) had grade 3 thrombocytopenia. Severe non-hematologic toxicities included two patients (3.0%) with grade 3 vomiting and one (1.5%) with pancreatitis. Toxicities were minimal during CCRT, with 4 patients (6.1%) showing grade 3 radiation-related mucositis and 1 (1.5%) with grade 3 radiation-related dermatitis. There was no treatment-related death.

**Table 2:** Toxicity observed during therapy

Toxicity	Grade1 (N. %)	Grade 2 (N. %)	Grade 3 (N. %)	Grade 4 (N. %)
**Hematological toxicity** Anemia Leukocytopenia Thrombocytopenia	17 (25.7%) 20(30.3%) 14(21.2%)	7(10.6%) 12(18.2%) 6(9.1%)	1(1.5%) 6(9.1%) 2(3.0%)	0 5(7.6%) 0
**Non-hematological toxicity** Nausea Vomiting Diarrhea Hepatic dysfunction	32(48.5%) 22(33.3%) 2(3.0%) 2(3.0%)	4(6.1%) 5(7.6%) 0 1(1.5%)	0 2(3.0%) 0 0	0 0 0 0
Pancreatitis	1(1.5%)			
Skin test positive/Allergy	30(45.5%)			
**Radiation-related mucositis** **Radiation-related dermatitis**	18(27.3%) 20(30.3%)	37(56.1%) 34(51.5%)	4(6.1%) 1(1.5%)	0 0

## DISCUSSION

Radiotherapy and chemotherapy are both important for treatment of ENKTCL. Radiotherapy specialists agree that early or ‘up-front’ radiotherapy improves the ORR and OS. Meanwhile, early chemotherapy is important as well as radiotherapy, because distant metastasis is the main reason inducing treatment failure [7–9, 12, 13]. It is well known that ENKTL resistance to anthracycline, the mechanisms may be related to the high expression of P-glycoprotein by lymphoma cells [14]. Therefore, anthracycline-based treatment did not achieve satisfied effect. Some novel regimes containing non-anthracycline drugs such as L-asparaginase or pegaspargase, cisplatin, oxaliplatin, ifosfamide, methotrexate, etoposide, gemcitabine and dexamethasone showed superior effect in ENKTCL [7–9]. However, the optimal regimen is still undefined. The protocols suggested by National Comprehensive Cancer Network Clinical Practice Guidelines include concurrent chemoradiation therapy (CCRT), sequential chemoradiation, sandwich chemoradiation and RT alone for patients unfit for chemotherapy. Some clinical trials including our previous research resulted [10, 15, 16] that “sandwich” chemoradiation was a promising protocol. We carried out a new regimen which was LVDP combined with CCRT, the “sandwich” protocol, in I/II stage patients aimed to discuss the optimal treatment. To our knowledge, our study is the largest prospective study regarding to treatment of newly diagnosed, I/II stage ENKTCLs.

**Figure 3:**
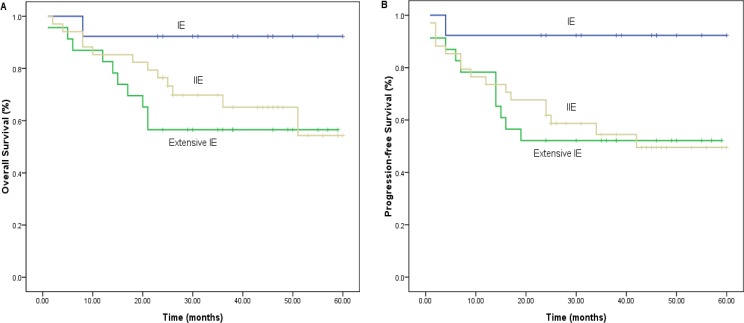
Analysis of prognostic value of Ann Arbor staging A. (for OS) and B. (for PFS).

In this study, 66 patients with stage I/II were enrolled. At the end of therapy, CR was 83.3%, PR was 3.0% and the ORR was 86.4%. The median follow-up was 23.5 months, with 3-year PFS of 67.4%, and 3-year OS of 70.1%. The median OS has not been reached. Compared with several previous prospective clinical studies focused on I/II stage ENKTCL patients [7–10] (Table 3), our results showed that short term response was similar with others. However, either 3-year OS or 3-year PFS was inferior. Through detailed comparison, we found that this difference may be due to: 1) the previous four studies had far smaller sample size than our study, which were in greater likelihood of bias; 2) The survival of patients with stage II and extra-cavity stage I were significantly worse than that of patients with limited stage I, while the survival difference between stage II and extra-cavity stage I was not statistically significant (Figure 3A and 3B). In present study, the patients with stage II and extra-cavity I accounted for 78.8%. However, the above-mentioned study carried by Wang et al. [7], Kim et al. [8] and Yamaguchi et al. [9] did not subdivide the stage I patients into limited I and extra-cavity I, with the phase II patients accounting for 33%, 50% and 33%, respectively. Even in our previous research, the proportion of extra-cavity phase I and phase II was only 50% [10]. This indirectly indicated that Ann Arbor stage system did not guide a better stratification for ENKTCL patients' survival.

**Table 3:** Clinical trials regarding to first-line chemoradiotherapy in I/II stage NK/T lymphoma

Study	Staging	No.of patients	Treatment regimen	ORR (%)	CR (%)	OS (%)	PFS (%)	Grade 3/4 toxicity (%)
Wang et al. [15]	IE=18 IIE=9	27	GELOX plus radiation	96.3	74.4	86(2-y)	86(2-y)	Hematologic 33.3 Mucositis 15
Kim et al. [16]	I=15 II=15	30	Concurrent chemoradiotherapy plus VIPD	100	73.3	86.3(3-y)	85.2(3-y)	Neutrocytopenia 40 Nausea 3
Yamaguchi et al. [17]	IE=22 IIE=11	27	DeVIC plus radiation	81	77	78(2-y)	67(2-y)	Leukocytopenia 97 Neutrocytopenia 91
Jiang et al. [18]	IE=13 Extended IE =7 IIE=6	26	LVP plus radiation	88.5	80.8	88.5(2-y)	80.6(2-y)	Leukocytopenia 7.7 Mucositis 23.1
This study	IE=14 Extended IE =29 IIE=23	66	VDLP plus Concurrent chemoradiotherapy	86.4	83.3	70.1(3-y)	67.4(3-y)	Leukocytopenia 16.7 Thrombocytopenia 3.0

ORR, objective response rate; CR, complete response; OS, overall survival; PFS, progression-free survival.

In addition, there are many other factors affecting prognosis. JY Lee and colleagues concluded NKIPI model composed of ‘B’ symptoms, stage, lactate dehydrogenase (LDH) level and regional lymph nodes, which could better predict prognosis [11]. SJ Kim et al. developed PINK model consisted of four risk factors (age, stage, non-nasal type, and distant lymph-node involvement) that were significantly associated with OS and PFS in ENKTCL [17]. In recent years, peripheral blood EBV-DNA copy number has been considered as a prognostic factor and related to therapeutic effect of ENKTCL patients [18, 19]. And now it is a routine laboratory test item at diagnosis, during treatment and following-up. Due to the limitation of laboratory conditions, it was tested in a small number of patients who were included in the late period of this study. Therefore, it was not involved in this report. However, the research about prognostic factors and prognosis system is in progress, and the results would be reported in subsequent paper.

Radiotherapy plays an irreplaceable role for cancer treatment. Usually, increasing the dose of radiotherapy might improve efficacy, while the radiotherapy-associated complications are increasing as well. For ENKTCL, the radiation dose is controversial. We reviewed literatures and found that it was variety in different clinical center, the range was from 40 to 65Gy [8, 13]. In the study of Wang H et al, the prescribed dose of 50Gy was planned with a boost of 5-10Gy to the residual primary disease, while the dose which the patients actually received was from 49.6 Gy to 64Gy, mean dose was 55.5Gy [20]. Our previous study suggested that dose of 56 Gy achieved local-control rate and toxicities were tolerated [10]. In this study, we continued to execute our previous radiotherapy protocol, it also showed good local control and was tolerated.

In present study, the total failure rate was 33.3% (22 cases), with 4.5% for local failure and 28.8% for systemic failure. Although they received second-line treatment, their median OS was only 15 months, which was far short compared with the whole patients' OS (23.5 months). In other words, the majority of failure was systemic that caused poor OS. This consisted with the other researches' results that most patients died soon after disease progression [7–9, 12, 13]. In addition, no matter using radiotherapy alone or CCRT, the rate of local failure was low and could be remedied. Compared with literature reports [7–10], although cisplatin was used to sensitize the radiotherapy, the benefits of local control and survival were not observed in this study. Therefore, the efficacy of systemic therapy was a significant factor impacting prognosis of I/II stage ENKTCLs. The value of CCRT still needs future research.

In this study, both hematologic and non-hematologic toxicities during therapy were lighter than reported results [7–9] and heavier than LVP [10]. But they were well tolerance. Pancreatitis caused by L-ASP should be worth paying more attention, because it can lead to seriously consequence, even death. In this study, one patient suffered acute pancreatitis in the third cycle of chemotherapy. After treatment, the patient's condition got improved and was still alive in disease-free situation at the end of following up. Through comprehensive assessment, we found that he had a history of chronic pancreatitis with significantly elevated serum triglyceride level. Therefore, light diet is recommended during therapy, especially in patients with a history of pancreatic disease or elevated blood lipids.

In summary, for the treatment of I/II stage ENKTCLs, the “sandwich” protocol is worthy of being generalized and LVDP combined with CCRT is an effective regimen with well tolerated toxicity. But this regimen was not suitable for all phase I/II patients and the value of CCRT needs further research. Therefore, in future studies, we will carry out larger sample and layering study for first-line therapy. In addition, another limitation about this study is that it was a single-institutional study. So a multi-centric collaborative study will be needed.

## PATIENTS AND METHODS

### Inclusion criterion

1) The ENKTCL patients were diagnosed based on pathological histology and immunohistochemistry, meeting the WHO criteria. 2) The patients with I/II stage. 3) Primary tumor located in the upper aerodigestive tract. 4) The patients received no radiotherapy or chemotherapy for ENKTCL. 5) White blood cell (WBC) ≥ 3,500/ul, absolute neutrophil count (ANC) ≥ 1,000/ul, platelet (PLT) ≥ 75,000/ul, glutamic-pyruvic transaminase (ALT) and glutamic oxalacetic transaminase (AST) < 2.5 times the upper limit of the normal level (ULN), total bilirubin < 2.0 mg/dL, creatinine clearance (Cr) ≥ 50 ml /minute. 6) Eastern Cooperative Oncology Group (ECOG) score of 0 to 2. 6) Expected survival time > 3 months. 7) Patients voluntarily participated and signed the informed consent.

### Exclusion criteria

1) Women during pregnancy or lactation. 2) Any clinical problems beyond our control (such as serious mental, nervous, cardiovascular and respiratory system diseases). 3) The researchers considered that the patient is not appropriate to participate.

This study was registered inwww.chictr.org.cn at 5/30/2012 and approved by the Chinese Ethics Committee of Registering Clinical Trials. The registration number: ChiCTR-TNC-12002353. The research was carried out in West China Hospital, Sichuan University. The methods were carried out based on the protocols recommended by National Comprehensive Cancer Network Clinical Practice Guidelines (NCCN Guidelines) and metabolic pathways of drugs ***in vivo***.

### Protocol

**Figure 4:**
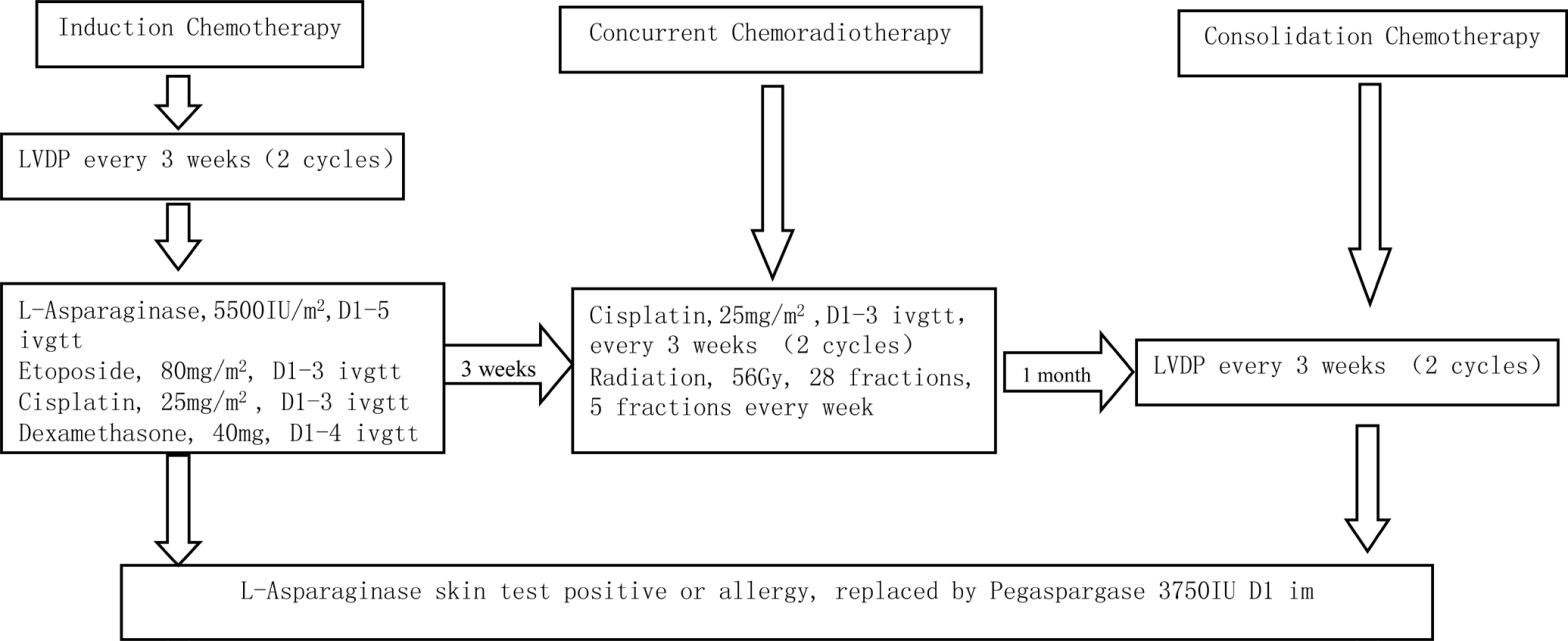
Treatment protocol

The treatment scheme was shown in Figure 4. Chemotherapy was started within 7 days after registration. The drugs' doses and administration schedule were as follows: L-asparaginase 5500 IU/m2 given intravenously on days 1 through 5, etoposide 80mg/m2 given intravenously on day 1 through 3, cisplatin 25 mg/m2 given intravenously on days 1 through 3, and dexamethasone 40mg/day given intravenously on days 1 through 4. The enrolled patients first received 2 cycles of LVDP as induction chemotherapy, followed by CCRT with radiation and 2 cycles of cisplatin alone. A month later, 2 cycles of LVDP were administrated for consolidating. Before using L-ASP at each cycle, skin test was performed. If the skin test was positive or the patients appeared allergic reaction caused by transfusing L-ASP, it was replaced by spegaspargase (3750 IU, D1) with intramuscular injection at three sites.

Routine blood test (RBT), liver and kidney function were tested before each cycle. The patient was not allowed to enter the next course until ANC ≥ 1,000/ul, PLT ≥ 75,000/ul and ALT and AST < 2.5 times of ULN. One to two RBT were performed every week during chemotherapy intermission. Granulocyte colony-stimulating factor (G-CSF) was administered if the patient developed grade 3/4 neutropenia. Grade 4 leukopenia or neutropenia happened first after chemotherapy, G-CSF was prophylactic administered in the following cycles. If grade 4 leukopenia or neutropenia happened again or grade 4 thrombocytopenia appeared, the dose of cisplatin and etoposide were reduced to 75% of initial dose during subsequent cycles. If 3/4 grade of toxicity persisted > 2 weeks, the patient was excluded.

Radiotherapy was scheduled after 2 cycles LVDP. Intensity modulated radiation therapy (IMRT) or three-dimensional conformal radiation therapy (3D-CRT) was used. Radiotherapy targets included gross tumor volume (GTV), clinical tumor volume (CTV) and planning target volume (PTV). GTV are the lesions evaluated by imaging and clinical examination. CTV is defined as the entire anatomical structure where the tumor infiltrates (CTV1) and the adjacent tissue structures where the tumor may get involved (CTV2). PTV is 3-5mm wider than GTV or CTV in three dimensions. The dose of GTV or CTV1 was 50-56Gy and CTV2 was 45-50.4Gy. Stage II patients whose primary site was nasal cavity and Webster ring needed prophylactic bilateral neck irradiation, with the dose of 45Gy. The irradiation was given in routine dose fraction, namely daily dose was 1.8-2.0Gy, one time a day, five times one week. Radiotherapy was delayed in patients with the following situations: grade 4 leukopenia or neutropenia; PLT < 25,000/uL; any grade 3 mucositis and dysphagia associated with radiation; ECOG score ≥ 3. Radiotherapy would be hold until the toxicity resolved to≤ 2. If the grade 3/4 toxicity persisted > 2 weeks, the protocol was terminated.

### Evaluation

Two weeks prior to the first cycle, the patients would receive a comprehensive evaluation, including complete medical history, physical examination, BRT, serum biochemistry test, electrolytes, lactate dehydrogenase (LDH), electrocardiogram, position emission tomography-computed tomography (PET-CT) or enhanced computed tomography (CT) or magnetic resonance imaging scanning for nasopharyngeal region and enhanced CT scanning for neck, chest and abdomen-pelvis, and nasopharyngeal laryngoscopy. The above examinations were repeated in one month after CCRT, one month after consolidated LVDP, and every 3-6 months during follow-up.

### Assessment

According to non-Hodgkin's lymphoma Efficacy Evaluation Criteria [21], treatment response was assessed. ORR included CR and PR, while SD and PD were judged to be invalid. OS defined as the time from enrollment to the end of follow-up or death, and PFS defined as the time from enrollment to the end of follow-up or discovery of disease progression or relapse. Definition and grading standards of adverse reactions were referred to the Common Terminology Criteria for Adverse Events version 3.0 developed by American National Cancer Institute.

### Statistical analysis

SPSS 16.0 (SPSS Japan Inc, Tokyo, Japan) was used. Kaplan-Meier method was used to calculate PFS and OS. Survival curves were compared by Log rank test. ***P*** value of < 0.05 was considered statistically significant.
